# Supplementary material for: Technical determinants of success in professional women’s soccer: A wider range of variables reveals new insights
Source: PLoS One. 2020 Oct 22;15(10):e0240992. doi: 10.1371/journal.pone.0240992 (PMC7580913; doi:10.1371/journal.pone.0240992)
Supplement: Attachment — Submitted filename: Response to Reviewers.docx [file pone.0240992.s001.docx]

**Comments to the Author**

1. Is the manuscript technically sound, and do the data support the conclusions?

The manuscript must describe a technically sound piece of scientific research with data that supports the conclusions. Experiments must have been conducted rigorously, with appropriate controls, replication, and sample sizes. The conclusions must be drawn appropriately based on the data presented.

Reviewer #1: Yes

Reviewer #2: Yes

*Authors: No response.*

2. Has the statistical analysis been performed appropriately and rigorously?

Reviewer #1: Yes

Reviewer #2: Yes

*Authors: No response.*

3. Have the authors made all data underlying the findings in their manuscript fully available?

The [PLOS Data policy](http://www.plosone.org/static/policies.action#sharing) requires authors to make all data underlying the findings described in their manuscript fully available without restriction, with rare exception (please refer to the Data Availability Statement in the manuscript PDF file). The data should be provided as part of the manuscript or its supporting information, or deposited to a public repository. For example, in addition to summary statistics, the data points behind means, medians and variance measures should be available. If there are restrictions on publicly sharing data—e.g. participant privacy or use of data from a third party—those must be specified.

Reviewer #1: No

Reviewer #2: No

*Authors: The following new paragraphs have been added to the cover letter.*

*“The data that was used for this study, was obtained from Opta Sports (*[*www.optasports.com*](http://www.optasports.com)*). The data was provided under a license agreement with Opta, and the data is also subject to an approved research ethics application from our University. The terms of the license agreement prevent us from sharing the raw data we used for this analysis. Our ethical approval also prevents us from sharing any data in any way that could be re-identified. The metadata and the (score) data itself would allow someone else to re-identify teams and possibly players. Releasing all or some of the raw data to PloS One, would breach our obligations to Opta Sports and our University research ethics committee.*

*However, anyone can contact Opta Sport and request the same dataset that we used in this analysis. We provide enough information in the methods and results sections, for other researchers to know what data to request from Opta Sport. We hope this explanation clarifies our position. If there are unresolved issues about data access, we would welcome the opportunity to discuss them with you.”*

4. Is the manuscript presented in an intelligible fashion and written in standard English?

PLOS ONE does not copyedit accepted manuscripts, so the language in submitted articles must be clear, correct, and unambiguous. Any typographical or grammatical errors should be corrected at revision, so please note any specific errors here.

Reviewer #1: Yes

Reviewer #2: Yes

*Authors: No response.*

5. Review Comments to the Author

*New line numbers reflect the edited manuscript with track changes when viewed in Word with “all markup”.*

**Reviewer #1:**

This is an interesting paper assessing the determinants of match outcome in élite women football. The paper is fairly well structured and conceived. The methodological approach is sound and nicely explained through informative pictures. The discussion is relevant and provides interesting insights.

*Thank you for these kind words and your time reviewing the manuscript.*

ABSTRACT

rows 16-21: please summarise previous research more succinctly.

*This part of the abstract has been made more concise.*

Line 21: tautology, “determinants” are “important” by definition.

*Now line 23. “Important” has been removed.*

Line 22: “analytical approached”, there’s something unclear in the sentence. Please define the sample size precisely (not “relatively large sample”).

*Now line 24. The grammar has been corrected and the sample size has been specified.*

Line 28: it is unclear what is being predicted.

*Now line 30. Amended to “in predicting match outcome”.*

The last sentence seems a repetition of what stated before.

*This has been edited to clarify we are referring to men’s soccer (football).*

INTRODUCTION

Line 41: in my opinion, as you’re talking about women football, it’s not necessary to highlight the drawbacks of studies on male players. Stating that such studies on women don’t exist could be a strong enough reason to justify the research.

*We are pleased that you think the analysis is justified, based on the limitations of research in women’s soccer alone. However, we wanted to achieve something more valuable than addressing this limitation alone. We felt that our large sample size (both number of team performance and the number of technical variables) presented an opportunity to contribute to future analyses in men’s and women’s soccer. We think our findings justify this claim.*

Line 55: the classification “accuracy”, “power”, etc. A word should follow “classification”.

*Now line 74. Added the word “accuracy”.*

Lines 60-61 are unnecessary and can be removed.

*Now line 78. Removed.*

Line 62: an “s” is missing after “relate”

*Now line 82. An “s” is added.*

Line 78: please add a space before “m” (meters)

*Now line 48. Space added.*

Line 92: why “understand” has capital letter?

*Now line 97. Amended.*

METHODS

I would call the first paragraph “data collection”

*This has been amended.*

An explanation of the variables domain (it’s not feasible to describe every one of the 450 variables) would be strongly advised.

*Line 111. A reference for description of the variables has been added in “Data Collection”.*

Line 105: so, if I get it right, you should have 266 variables per each team per each game, right? If so, this should be explained more clearly.

*Now line 110. Yes that is correct and we have clarified this in that sentence.*

Line 105: “variables without values in them” awkward expression.

*Now line 109. Changed to “empty variables”.*

Line 107: data normality testing should be explained in the data analysis paragraph.

*You are right. We have moved these statements to the “Analysis section”, line 128.*

Line 109: there’s no need to explain what R is.

*Now line 116. We have simplified the in-text referencing to the reference number only.*

Please remove the “tables” heading.

*It has been removed.*

Line 138: why did you keep 152 variables? Which was the criterion?

*Now line 145. We removed variables that were too closely related to goal scoring. In other words, variables (actions) that almost always precedes a goal. We have clarified this in the manuscript, line 154.*

Line 159: how did you choose the correlated variable to remove?

*Now line 169. We provide a reference for this process (33). For any pair of variables that exceed the r>0.90 correlation threshold, the variable with the higher mean correlation with all other variables is removed. For the sake of conciseness, we would prefer not to expand on this process in the manuscript.*

Line 188: please remove “was utilised”

*Now line 198. If this is removed the sentence does not have a verb and would be grammatically incorrect.*

DISCUSSION

Line 255: is there anything related to women’s specific psychology in that? I mean, could it be that women when trailing by one goal, could more easily demoralised and struggle to score? Try to clarify / comment this point.

*Now line 267. It is difficult to explain this effect but we have added speculation that there may be a psychological aspect to the effect.*

Line 266: just a comment, GK proficiency is probably even more important in women soccer for physical (stature) reasons. Thus, disposing of a tall, athletic and skilled keeper (i.e. one able to save a lot of shots) is a competitive advantage for women (relatively more than in men).

*That’s an insightful observation, thank you.*

Line 321: that’s true. Why wasn’t it done asking coaches to select variables? Have the Authors themselves first-person experience in women’s football.

*Now line 312. None of the authors have experience coaching women’s soccer, although most of the authors have watched closely and studied soccer for many years. We did not have local access to an elite women’s coach that would be familiar with all the variables. Asking one coach has its own limitations and then asking several coaches obliges us to use something like the Delphi method which would add unnecessary complexity to the report. In any case, we have conceded this as a limitation of this study.*

Line 328: please remove the comma after “analysis”.

*Now line 324. Coma has been removed.*

The last sentence could be removed painless.

*We disagree because we think this point needs to be emphasised for novice analysts.*

REFERENCES

Please double check references for consistency (ie. n. 6 but not limited to)

*All references have been double checked and errors have been corrected.*

**Reviewer #2:**

General Comments to Author
The authors used three different approaches to identify important technical features/variables in women’s soccer. The authors investigated a high number of variables and a large sample size, which is a major strength of the present manuscript.

*Thank you for these words of appreciation and your time reviewing this manuscript.*

Specific comments

INTRODUCTION

In the introduction authors focus primary on studies in men’s soccer and the corresponding limitations (lines 39-69). While the comparison of men’s soccer and women’s soccer is of additional interest, the primary focus of the present manuscript should be on studies in women’s soccer and the corresponding limitations (as stated in the aims of the study in lines 92-95). The reviewer suggests shifting the focus on studies in women’s soccer und to reformulate the corresponding sections.

*Thank you for your observation. We agree that the primary purpose and application of this study relates to advancing our understanding of women’s soccer. So, we have restructured the introduction to present the content about women’s soccer first. The secondary aim of the study relates to methodological issues that may apply to analysis in both women’s and men’s soccer. However, to describe the background and rationale for this topic, we have to rely on references from men’s soccer as there are so few studies of women’s soccer. We have adjusted the wording of these paragraphs so that they appear to be less of a criticism of studies of men’s soccer specifically, and more generally about methods.*

MATERIALS AND METHODS

Line 105: Please insert the number of variables that were removed (n=10?).

*Now line 112. Have added the number of variables (n = 3) that were removed due to being empty.*

Line 140: At this point it is unclear to the reviewer how the variables relevant to coaches were chosen. As mentioned in the discussion section, variables were not chosen based on coaches feedback. Why?

*Now line 152. None of the authors have experience coaching women’s soccer, although most of the authors have watched closely and studied soccer for many years. We did not have local access to an elite women’s coach that would be familiar with all the variables. Asking one coach has its own limitations and then asking several coaches obliges us to use something like the Delphi method which would add unnecessary complexity to the report. In any case, we have conceded this as a limitation of this study*

Line 147: Please be more specific what a “nearly direct relationship” to goal scoring mean. For example, ‘shots on goal’ were excluded while ‘intentional assists’, which result in shots on goal, were excluded.

*Now line 154. We removed variables that were too closely related to goal scoring. In other words, variables (actions) that almost always precede a goal. We have clarified this in the manuscript.*

Lines 150: Please reformulate “more trainable aspects”, because it is not consistent with ‘shots on goal’ (line 148) for example.

*Now line 158. We understand your concerns about the consistency between “more trainable aspects” and “shots on goal”, so we have changed the wording and the point we make. Psuedo-score related variables dominate the models we create, which confirms their importance but also “obscures” the importance of other variables.*

Line 159: In the reviewer’s opinion, authors should check these excluded variables. For example, if the features selection process identifies an important variable, which has a high correlation (r>0.9) with one or more of the excluded variables, these excluded variables might also be important.

*Now line 169. We understand your concern. There is a higher risk associated with retaining these variables. By retaining several collinear variables, they will dominate the model without adding new information. Their domination will also eliminate other different variables that add new information.*

Line 168: Please be more specific with respect to “reduced model accuracy” because a higher number does not necessarily reduce model accuracy. For example, phase 1 resulted in higher accuracy than phase 2 where lower variables were included.

*Now line 178. Including too many variables in the model training process can add noise to the model and/or cause overfitting. Both of which, reduce subsequent model accuracy in the validation phase. We have clarified this in the manuscript.*

Line 174: Please specify “MA”, because it was not specified before.

*Now line 184. Apologies, “MA” was the abbreviation of the previous name we used for the Literature Driven Approach (LDA). It has been replaced with LDA.*

Line 177: Table 2: A reader of the manuscript might benefit from a more detailed description of the listed variables. If possible, the reviewer would suggest providing a more detailed description as supplementary information.

*Now line 187. The publisher of the data provides a comprehensive list of definitions online. A reference for the events has been added, line 110.*

RESULTS

Line 197: Table 3: In Table 3 the listed variables correspond to the GLM model only. This should be indicated somewhere.

*Now line 220. The most left column labels the variables in these rows as “GLM predictors”.*

Line 203: Please reformulate “slightly higher than random choice”.

*Now line 213. Changed to “slightly higher than a 50% chance of win/loss”*

Line 206: Table 3 might be more appropriate than Table 2.

*Now line 216. This has been changed to “Table 3”.*

Line 208: Please insert almost or another alternative before equally since the models did not perform equally.

*Now line 218. The word “almost” has been inserted.*

Lines 215-227: As noted above it should be noted that the specific variables correspond to the GLM models. Only in lines 235 to 237 specific variables corresponding to the DT approach are presented. Why did the authors decide to exclude a more detailed description of the specific variables of the DT approach in the manuscript?

*A DT does not provide coefficients that represent the relative importance of the variables. Even ranking the variables is not always possible as there may be variables at a similar level within the tree, on different paths. The results of the GLM provide ranking and the magnitude of importance. We decided that the best combination of the two analyses was to use the GLM to understand relative importance of the variables and the DT to communicate interactions between, and critical values of the variables.*

Lines 231-232: A reader might be interested in the difference between “intentional assists” and “normal assists”. As mentioned above a more detailed description would be beneficial and should be provide as supplementary information, if possible.

*A reference to Opta Sport’s event definitions has been added, line 110.*

DISCUSSION

In the first paragraph should refer to the two objectives (aims) of the study stated at the end of the introduction (i.e., understanding the technical determinants of success in women’ soccer (a) and analytical approaches (b)). Although the comparison to men’s soccer is of interest, it should be discussed in a later paragraph.

*Now line 258. The focus of this paragraph has been shifted toward the two aims.*

Lines 261-264: Because an intentional assists results in a shot on goal, one might also infer that shots on goal preceding an intentional assist are more important than shots on goal in general. Please consider this.

*Now line 276. We have added a sentence to concede this point. “However, given that shots on goal precedes assists, it is difficult to definitively separate the importance of these two actions.”*

Line 274: Please reformulate “offensive style of play”, because passes refer more to ball possession and (aerial) duels might refer more to the physical condition (i.e., sprinting and jumping abilities). These characteristic might be well addressed in training as well.

*Now line 288. We have reworded this interpretation to have less of a focus on “offense” and “technical”. The wording of the interpretation has been broadened to cover both offence and defence as being “aggressive”.*

Line 290: Please consider that incorporating relative scores might also be important.

*Now line 305. If you are suggesting that relative match score is important for modelling, we disagree. The range of relative score in soccer is much smaller than for other related sports, and therefore it doesn’t represent relative team performance very well, nor does it make a mathematically valuable target variable for modelling.*

Lines 291-303: Please move this paragraph, because recommendations for future research are typically given at the end of the manuscript.

*Now line 327. This and the following paragraph (which also contains recommendations) have been moved to the end of the discussion section.*

Lines 313-314: Please reformulate the example, because “seeking 2 more assists than their opponent” is not applicable.

*Now line 348. We are not sure what you mean by “applicable”. If you mean that this is hard to practically implement, then we agree – this is the point we are making in this sentence. We have adjusted the wording to clarify the difficulties associated with this.*

Lines 317-319: Did the authors look at the results for a subset of the data to support this hypothesis?

*Now line. 344. No we didn’t. The statement is provided here as mere speculation about a plausible possibility. A clarification has been added to the sentence.*

Lines 320-322: See comment above (Line 140).

*We have responded to this comment above.*

Lines 315-322: Please consider in the limitations also the following points. First, technical indicators are not the only determinants of success. For example, physical aspect, tactical decisions or psychological factors are also import. Second, please consider also the problem of stability of feature selection (i.e., in the presence of correlations).

*Now line 314-318. We have added sentences to concede both points.*

END
