# Supplementary material for: Technical determinants of success in professional women’s soccer: A wider range of variables reveals new insights
Source: PLoS One. 2020 Oct 22;15(10):e0240992. doi: 10.1371/journal.pone.0240992 (PMC7580913; doi:10.1371/journal.pone.0240992)
Supplement: Attachment — Submitted filename: Response to Reviewer #2.docx [file pone.0240992.s002.docx]

**Response to comments Reviewer #2**

We thank the reviewer for their final comments about the introduction.

We do not want to jeopardise the opportunity we have to publish our manuscript, especially at this late stage of the review process. If the reviewer’s feedback were simple to implement and didn’t have any unintended consequences, we would certainly comply with their suggestions. However, we find the reviewer’s suggestions are difficult to implement, because they would weaken the development of the rationale for the study. We are also concerned the changes would be substantial and that the other reviewer, who has approved the introduction, may not approve of a substantially different version.

The main theme of the reviewer’s query relates to why we include information about men’s football, in a manuscript that is based on data from women’s football. There are two main reasons for this;

1) There is only one study in women’s soccer that solely focusses on technical performance related to success that used more than a single variable. The three other studies mentioned in the manuscript either use one variable (scoring first or free kicks) or have an aim that is not focussed on technical performance, but they include some variables that could be considered technical. So, there is very little opportunity for us to fill the introduction with a summary of these articles if we removed the references to men’s football.

2) The second aim of the study is about potential improvements in analytical approaches used in technical analysis in soccer, regardless of the gender. As such, there is a need to summarise the methods that have been used in related articles in men’s football. We are not trying to discredit previous analyses of technical analysis in men’s football. We are simply trying to provide a critique of relevant papers and their methodological issues, almost all of which are about men’s football. Consequently, the introduction is dominated by references to relevant studies of men’s football.

If we remove the content in the introduction that relates to men’s football, we will weaken the rationale for our aims and we also remove the background information that is necessary for the discussion. The reviewer has suggested that we could include “limitations of studies on men’s soccer” in the fifth paragraph. This is the final paragraph of the introduction, where we need to summarise the main points, clarify the rationale, and state our aims. We don’t think it would be appropriate to introduce a critique of analytical methods here.

As compromise, we provide edits to the introduction that make some of the points that appear above and address some of the reviewer’s specific comments. We have adjusted the structure of some paragraphs to declare their purpose more clearly and remove perceptions of repetition. More specifically, we decided to merge line 51-53 (limited sample size and number of variables in women’s soccer) to the separate paragraphs about these separate methodological limitations regardless of gender. Also, regarding the reviewer’s comment about the focus on statistical models in men’s soccer, the limitations are also valid for some of the women’s studies, so we added the women’s references to that argument. Please see the document with track changes for the exact details of the revisions.
